# Validation and Clinical Application of a Liquid Chromatography-Ultraviolet Detection Method to Quantify Dolutegravir in Dried Blood Spots

**DOI:** 10.1097/FTD.0000000000000929

**Published:** 2022-06-01

**Authors:** Abdulafeez Akinloye, Oluwasegun Eniayewu, Babatunde Adeagbo, Oluseye Bolaji, Adeniyi Olagunju

**Affiliations:** * Department of Pharmaceutical Chemistry, Obafemi Awolowo University, Ile-Ife, Nigeria; † Department of Pharmaceutical and Medicinal Chemistry, University of Ilorin, Ilorin, Nigeria; ‡ Department of Pharmacology and Therapeutics, Institute of Systems, Molecular and Integrative Biology, University of Liverpool, Liverpool, United Kingdom

**Keywords:** Dolutegravir, Dried blood spot, Pharmacokinetics, HPLC-UV, HIV

## Abstract

**Background:**

Dolutegravir is currently the preferred component of first-line antiretroviral therapy. To facilitate clinical pharmacology studies in key populations, quantitative analytical methods compatible with microsampling and adaptable to resource-limited settings are desirable. The authors developed and validated a liquid chromatography-ultraviolet detection method to quantify dolutegravir in dried blood spots (DBS).

**Methods:**

Calibration standards and quality control samples were prepared by spotting 50 μL of dolutegravir-spiked whole blood on each circle of DBS cards. Three spots (two 6-mm punches/spot) were extracted with methanol. Chromatographic separation was achieved with gradient elution of acetonitrile/potassium phosphate monobasic buffer (pH 5) on a reverse-phase C18 column (flow rate, 1 mL/min) using pioglitazone as the internal standard. UV detection was performed at 260 nm. In the clinical pharmacokinetic study, DBS from finger prick was collected from participants (n = 10) at 8 time points over 12 h post-dosing, with paired plasma at 1 and 12 h. The method was used to quantify dolutegravir, estimating pharmacokinetic parameters. Agreement between DBS and plasma concentrations was evaluated using linearity and Bland-Altman plots.

**Results:**

The method was validated over the concentration range of 0.4-10 μg/mL, accuracy was 102.4-114.8%, and precision was 3.4-14.7%. The mean recovery was 42.3% (%CV: 8.3). The mean (±standard deviation) dolutegravir concentration in DBS was 37.5% (±3.8%) lower than that in the plasma. DBS-derived and measured plasma concentrations showed strong correlation with linearity (R^2^ = 0.9804) and Bland-Altman plots. Means (%CV) of AUC, C_max_, and C_24_ from the DBS-derived plasma concentration were 37.8 (23.2) μg.h/mL, 2.7 (24.7) μg/mL and 1.34 (31.6) μg/mL, respectively.

**Conclusions:**

The application of this simple, accurate, and precise method will expand opportunities for clinical assessment of dolutegravir in resource-limited settings.

## Introduction

Dolutegravir is a potent tricyclic carbamoyl pyridine second-generation integrase strand inhibitor approved by the US Food and Drug Administration as part of a combination first-line treatment in 2013.^1^ The 2019 World Health Organization updated treatment guidelines recommend it as the preferred component of first-line antiretroviral therapy (ART) in all human immunodeficiency virus (HIV)-infected population groups, including pregnant and breastfeeding women, except neonates.^2^ This update was based on accumulating data from multiple studies^3^ in which the original link between periconceptional dolutegravir use and neural tube defects was not detected.^4,5^ This, in addition to the previously reported higher potency, the higher genetic barrier to resistance, and better safety profile compared with other drugs, further supports the recommendation.^6,7^ Furthermore, less than 50% of patients receiving ART in 2017 were reported to achieve viral suppression (plasma HIV RNA < 1000 copies/mL). To achieve the third component of the Joint United Nations Programme on HIV/AIDS (UNAIDS) vision 90-90-90,^8^ introduction of more potent drugs like dolutegravir into first-line ART regimen became necessary.

Large-scale switching of patients to regimens containing dolutegravir is ongoing or planned in several countries worldwide. Several clinical pharmacology studies are anticipated, especially in pediatrics and pregnant women, including studies to investigate the influence of key covariates on disposition, development and assessment of new pediatric formulations, bioavailability and bioequivalence studies, and therapeutic drug monitoring (TDM). Ensuring the long-term efficacy of dolutegravir-based regimens will require optimal adherence. For instance, an estimated 20-35% of patients on ART are nonadherent, partly due to side effects experienced with efavirenz-based regimens.^9,10^ Therefore, TDM is an important strategy to sustain dolutegravir efficacy in patient populations with sub-optimal adherence. Several liquid chromatography tandem mass spectrometry (LC-MS/MS) or high-performance liquid chromatography with ultraviolet detection (HPLC-UV) methods have been reported to analyze dolutegravir in various matrices, including plasma,^11,12^ male genital tract,^13^ hair,^14^ cerebrospinal fluid,^15^ and cervicovaginal fluid.^6^ Several of these methods are associated with certain challenges that render them difficult to implement in settings with limited resources, including high instrumentation costs for LC-MS/MS or reliance on ultra-low-temperature facilities for storage and transportation. Studies in these areas often rely on HPLC-UV methods, given the affordability; however, they are often challenged with detecting analytes at markedly low concentrations, rendering drug assays at low concentrations difficult. Additionally, these methods can only be applied to UV active compounds or those that do not encounter interference from the use of other UV active reagents. Interest in the use of dried blood spots (DBS) for clinical pharmacokinetic studies has grown in recent years.^16^ Compared with venous blood collection, the DBS method is simpler, less invasive, has a smaller blood volume requirement, is cheaper, allows room temperature storage for most analytes, and easier transportation, as it is classified as a relatively low-risk biological material of Category B (UN3373).^17^ These are especially attractive to researchers in low-and middle-income countries.^18^ Furthermore, sampling can be self-performed by patients.^19^ It has been used to analyze other antiretrovirals,^20–22^ and was determined as a suitable alternative to plasma-based methods.

Herein, we report a simple DBS HPLC-UV method to quantify dolutegravir in individuals receiving dolutegravir-containing regimens. Additionally, the method was cross-validated with plasma analysis to evaluate its potential as an alternative sampling strategy for future clinical trials.

## Materials and Methods

### Materials

Dolutegravir sodium and pioglitazone were procured from AK Scientific (Union City, California, USA). Potassium phosphate monobasic was obtained from Burgoyne Burbridges (Mumbai, India), and acetic acid and formic acid were obtained from Sigma-Aldrich (St. Louis, MO, USA). HPLC-grade acetonitrile and methanol were obtained from Honeywell Riedel-de Haёn (Seelze, Germany). Distilled water was produced by a quartz double distillation unit (Bhanu Scientific, Karnataka, India) and filtered using MF-Millipore Membrane Filters (0.45 μm pore size; Merck, North-Holland, The Netherlands) using a filtration apparatus (VWR, Leicestershire, England). DBS cards (Whatman 903 Protein Saver) were purchased from GE Healthcare Life Sciences (New York, NY, USA), and safety lancets were obtained from BD (Oxford, UK).

### HPLC-UV Systems and Conditions

The Sykam chromatography system (Sykam, Fürstenfeldbruck, Germany) consisted of an isocratic and quaternary gradient S 1130 HPLC pump system (version 1.0), 100 μL S5300 autosampler/ injector (version 1.1), and an S 3250 UV/visible detector (version 1.1) with a wavelength range of 190–800 nm. The column used was a reverse-phase Eclipse XDB-C18 (5 μm particle size, 150 mm x 4.6 mm; Agilent Technologies, Santa Clara, CA, USA). Gradient elution (flow rate of 1 mL/min), consisting of 50 mM potassium phosphate monobasic at pH 5 (mobile phase A) and 100% acetonitrile (mobile phase B), was used for chromatographic separation. The gradient program started with 70% A, maintained for 2 min, reduced to 57.5% at 5 min. It returned to 70% at 11 min, followed by column equilibration to the initial conditions for 2 min. The total run time was 13 min. The detector was set at 260 nm, and separation was performed at room temperature.

### Stock Solutions, Calibration Standards and Quality Controls (QCs)

Dolutegravir sodium and pioglitazone (internal standard [IS]) stock solutions (1 mg/mL) were prepared from their reference standards in 100% methanol and maintained at -20°C until use.^23^ A whole blood (10 μg/mL) working stock of dolutegravir was prepared by spiking blank whole blood with 1 mg/mL stock solution, tumbling for 30 min to achieve adequate mixing. This was used to prepare six whole blood calibration standards ranging from 0.4–10 μg/mL by serial dilutions. This concentration range was selected to cover the anticipated concentration range of dolutegravir in clinical specimens.^6^ A separate whole blood working stock, prepared by spiking whole blood with 1 mg/mL dolutegravir stock solution, was diluted with drug-free whole blood to prepare the low concentration quality control (LQC; 0.5 μg/mL), medium concentration quality control (MQC; 4.5 μg/mL) and high concentration quality control (HQC; 8.0 μg/mL) samples. All calibration standards and QC samples were tumbled for 30 min to ensure adequate mixing. A 250 μg/mL pioglitazone solution in methanol was prepared from 1 mg/ml stock solution and used as an IS. DBS calibration standards and QC samples were prepared by carefully spotting 50 μL of whole blood calibration standards and QCs on each circle of Whatman 903 protein saver cards. Spotted cards were dried at room temperature overnight, packed in zip-lock bags with desiccant sachets, and stored at -70°C until analysis.

### Sample Pretreatment

Six DBS punches were generated from three spots (two 6-mm punches per spot) and transferred into a 7-mL extraction tube with a screwcap, known as spot stacking.^20^ After adding 980 μL of methanol and 20 μL (250 μg/mL) of pioglitazone, the tubes were gently tumbled for 1 h. The IS was added alongside the extraction solvent, as direct spotting onto the cards was not feasible for clinical application. The extract was transferred into a 5 mL tube and centrifuged at 14500*xg* for 10 min. The supernatant was then collected into another extraction tube and evaporated to dryness at 60°C. The stability of dolutegravir was evaluated at this temperature (see below). The extract was reconstituted in 500 μL of methanol, and 300 μL was transferred into autosampler vials following centrifugation at 14500*xg* for 10 min. The injection volume was 100 μL.

### Calibration Curves, Accuracy and Precision

Three different DBS assay batches consisting of a zero blank, six levels of calibration standards (n = 2 per level), and QCs (n = 6 per QC level) were run. Calibration curves were constructed using a linear regression equation of analyte/IS peak area ratio against the nominal concentration. Accuracy was defined as the percentage deviation of the measured concentration from the nominal value, and precision was defined as the percentage coefficient of variation (%CV). For every validation or clinical sample analysis batch, not less than 75% of all standards and 67% of all QCs were required to have a percentage deviation within ±15%.

### Recovery and Robustness of Extraction Procedure

The recovery of dolutegravir from DBS was evaluated by comparing the mean of peak responses from replicates of extracted DBS at each of the three QC levels (n = 5 each) with the mean of peak responses of QC samples prepared by spiking dolutegravir into extracted drug-free DBS (n = 5 each); the latter represented 100% recovery. DBS samples for the recovery experiments were prepared with blank whole blood collected from healthy volunteers at three hematocrit levels (0.2 L/L, 0.4 L/L, and 0.6 L/L).^24^ The blood samples that required plasma removal to achieve the increased hematocrit value were centrifuged at 3000*xg* for 5 minutes, followed by removal of required plasma. All blood samples were gently mixed for 30 min after the addition and/or removal of plasma. The hematocrit values were then determined to confirm target values. Blood samples were spiked with dolutegravir to obtain QC samples, and dried at room temperature for 3 h before analysis. To evaluate the effect of short-term storage on recovery, another set of QC samples (n = 5 per level/hematocrit value) was prepared and stored at 60°C for 72 h, after which dolutegravir was quantified and compared with freshly prepared samples.

### Specificity and Stability in Solution

The influence of interference from tenofovir and lamivudine, commonly co-administered with dolutegravir as part of fixed-dose combination tablets, was evaluated by injecting a mobile phase spiked with dolutegravir, tenofovir disoproxil fumarate, and lamivudine (2, 12, and 12 μg/mL, respectively). To assess the stability of dolutegravir in solution at different temperatures, 1 μg/mL working stock was prepared, and 1 mL aliquots were transferred into different 7 mL tubes and stored for 12 h at five different temperatures (n = 3 for each): −20°C (as control), 4°C, 25°C, 60°C, and 70°C. The temperature stability of dolutegravir solution in methanol at –20°C was previously reported^23^; hence, it was chosen as the control. Samples stored at 60°C and 70°C were dried and reconstituted in 1 mL of methanol. All samples were analyzed and compared in percentages with values obtained at −20°C.

### Plasma Analysis for Cross-Validation

A partial validation of the method to quantify dolutegravir in plasma was conducted. Plasma calibration standards ranging from 0.4-10 μg/mL (n = 2 per level) and QC samples (LQC, 0.5 μg/mL; MQC, 4.5 μg/mL; and HQC, 8.0 μg/mL; n = 6 per level) were prepared by spiking blank plasma samples from a healthy volunteer with dolutegravir. Calibration curves were constructed using a linear regression equation of analyte/IS peak area ratio against the nominal concentration. No less than 75% of all standards and 67% of all QCs in any batch were required to have a percentage deviation within ±15%.

Extraction of plasma samples (calibration standards, QCs, and patient samples) was performed by adding 150 μL of dolutegravir-spiked plasma and 30 μL IS, followed by 570 μL of acetonitrile to precipitate proteins. The mixture was briefly vortexed and left to stand for 10 min. The mixture was vortexed again, followed by centrifugation at 16100*xg* for 10 min. The supernatant (300 μL) was transferred into an autosampler vial for injection.

### Clinical Application

The method was employed in a clinical pharmacokinetic study involving recently postpartum HIV-positive women (n = 10) taking 50 mg dolutegravir once daily, 300 mg tenofovir disoproxil fumarate, and 300 mg lamivudine in a fixed-dose tablet formulation. The patients were part of a cohort study investigating the viral and antiretroviral dynamics in mother-to-child transmission fluids (VADICT study).^25^ Women were recruited from participating hospitals in Benue State, Nigeria (Federal Medical Center, Makurdi and Bishop Murray Medical Center, Makurdi), enrolled early in pregnancy and followed up until 12-18 months postpartum. The study protocol and amendments were approved by the National Health Research Ethics Committee, Abuja, Nigeria (NHREC/01/01/2007). All patients in the VADICT study were taking efavirenz-containing regimens at study entry and were allowed to remain in the study after switching to dolutegravir-containing regimens owing to treatment policy changes. Patients who participated in this pharmacokinetic sub-study were switched between 4 and 6 weeks before pharmacokinetic sampling.

Briefly, DBS samples were collected from each patient at 0.25, 0.5, 1, 2, 3, 6, 12, and 24 h following an observed dose of dolutegravir-containing regimen (taken after a standard local meal), using a finger prick with a 2 mm safety lancet after sterile cleaning with isopropyl alcohol. At each time point, the first drop of blood was wiped clean, and subsequent blood drops were collected on the five circles of the DBS card. To facilitate direct comparison and cross-validation of dolutegravir concentration obtained from DBS with plasma, paired DBS and plasma samples were collected at the 1-h and 12-h time points. All DBS samples were dried at room temperature for 3-4 h, packed with desiccant sachets in zip-lock bags for storage at room temperature, and analyzed within 3 weeks. Six DBS punches, generated from three spots (two 6-mm punches per spot), were extracted for each sample. Plasma samples were stored at –80°C until transfer in an Arctic Express^®^ Dry Shipper (Thermo Scientific, Waltham, MA, USA) to the Translational Pharmacokinetic Research Laboratory, Faculty of Pharmacy, Obafemi Awolowo University, Nigeria, and stored at −70°C until analysis.

### Data Analysis

Clarity (DataApex, Prague, Czech Republic) was used for HPLC data processing. The concentration-time data from patients were processed with GraphPad Prism, version 5.01 (GraphPad Software, La Jolla, CA, USA) using non-compartmental analysis to obtain pharmacokinetic parameters at 90% confidence interval (CI), including area under curve (AUC), maximum concentration (C_max_), peak time (t_max_), and trough concentration (C_24_). DBS-derived plasma concentration was obtained from DBS concentration using the formula, [DBS_conc_/(1 - HCT)]x0.99, where DBS_conc_ is the measured dolutegravir concentration in DBS, HCT is the hematocrit (published average of 0.40 L/L in women was used^26^ as the hematocrit effect is very low), and 0.99^6^ is the plasma protein-bound fraction of dolutegravir. Agreement between DBS-derived plasma and measured plasma concentration was evaluated using linear regression using Microsoft Excel and Bland-Altman analysis using GraphPad Prism.

## Results

### Chromatographic Conditions

**Figure 1:**
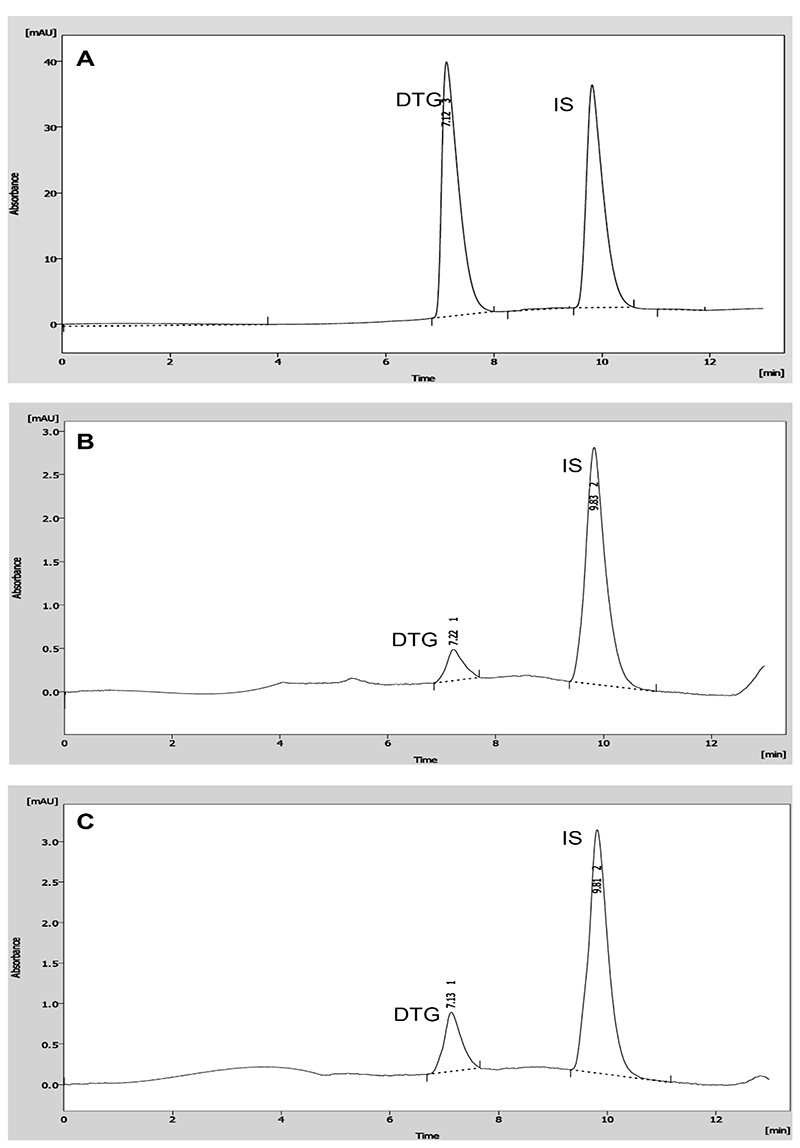
Representative chromatograms of (A) dolutegravir and internal standard (IS; pioglitazone) spiked in the mobile phase; (B) Lowest limit of quantitation (LLOQ; 0.4 μg/mL dolutegravir) extracted from DBS and IS, and (C) Low quality control (LQC; 0.5 μg/mL dolutegravir) extracted from DBS and IS. DTG, dolutegravir; DBS, dried blood spot.

Chromatographic separation was achieved using gradient elution with acetonitrile and phosphate buffer as the mobile phase. Retention times for dolutegravir and IS (pioglitazone) were 7.2 and 9.9 minutes, respectively; the total run time was 13 min. Representative chromatograms (Fig. 1) indicate that both peaks were baseline-resolved from endogenous components.

### Calibration Curve, Accuracy and Precision

The calibration curve of dolutegravir in DBS over the concentration range of 0.4–10 μg/mL was linear, the percentage bias (back-calculated concentration compared with nominal concentration) was consistently within 15%. A typical calibration curve equation was y = 0.8874x – 0.0152. The accuracy ranged from 85.24-112%, and the precision ranged from 3.4-14.67% (Table 1).

### Recovery and Robustness of Extraction Procedure

The average dolutegravir recovery from DBS was 42.26% (%CV = 8.3). There were no significant differences in dolutegravir recovery from DBS at hematocrit levels of 0.2-0.6 L/L at all QC levels, and %CV was within 15% at each hematocrit and QC level. Additionally, dolutegravir recovery from freshly prepared samples was comparable to recovery from samples subjected to accelerated short-term storage (60°C for 72 h) at all three QC levels, with hematocrit within 0.2-0.6 L/L (Table 2). This indicates the robustness of the extraction procedure.

### Specificity and Stock Solution Stability

The specificity of the method was evaluated to ensure that there was no interference from other drugs commonly co-administered with dolutegravir. This was evaluated by injecting a solution containing dolutegravir, lamivudine, and tenofovir disoproxil fumarate. The peaks of both drugs did not interfere with that of dolutegravir; the retention times of lamivudine and tenofovir were 2.2 and 6.6 min, respectively, when compared with 7.2 min for dolutegravir. Dolutegravir stock solution was stable at 4°C, 25°C, and 60°C after 12 h of storage when compared with the stability at −20°C. A lack of stability was observed at 70°C, owing to drug degradation.

### Cross Validation of DBS with Plasma Dolutegravir Concentration

**Figure 2:**
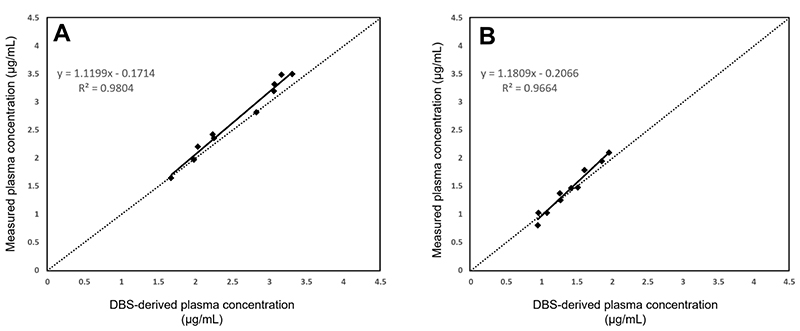
Linear relationship between DBS-derived plasma concentrations and measured plasma concentrations at (A) 1-h post-dose; and (B) 12-h post-dose. The diagonal line represents the true line of identity. DBS, dried blood spot.

A strong correlation was observed between plasma and DBS dolutegravir concentrations, especially at the 1-h post-dose time point (Pearson correlation coefficient (R^2^) = 0.9804). The DBS dolutegravir concentration was on average (±SD) 37.5 ±3.8% lower than the measured plasma concentration. The relationship between dolutegravir plasma concentrations (DBS-derived plasma concentration) calculated from DBS concentrations and the measured plasma concentration was evaluated using the closeness of the linear curve to the true line of identity and Bland-Altman plots. On evaluating the linear relationship between DBS-derived and measured plasma concentrations at 1- and 12-h post-dose (Fig. 2), a stronger positive correlation was observed between the concentrations at 1 h (R^2^ = 0.9804; y = 1.1199x - 171.42; n = 10), less close to the true line of identity than at 12 h (R^2^ = 0.9664; y = 1.1809x - 206.62; n = 10).

**Figure 3:**
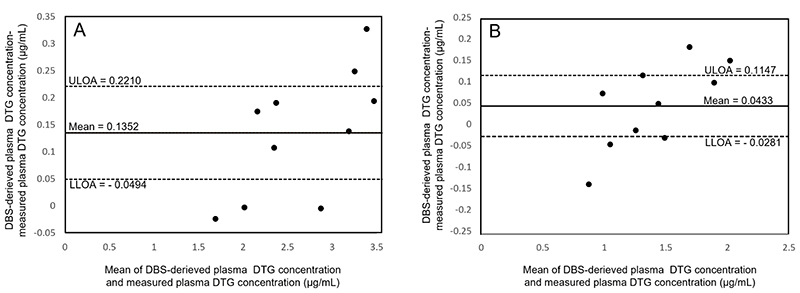
Bland-Altman plots for dolutegravir (A) 1-h post-dose and (B) 12-h post-dose for dolutegravir using mean hematocrit for females. The continuous line indicates the mean, and the broken lines represent 95% CI for the mean difference (±0.12 SD for A and ±0.10 for B). DBS, dried blood spot; DTG, dolutegravir; LLOA, lower limit of agreement; ULOA, upper limit of agreement; CI, confidence interval.

Bland-Altman plots of 1- and 12-h post-dose dolutegravir concentrations (Fig. 3) indicate the relationship between the difference and average of the DBS-derived and measured plasma concentrations obtained with a 95% CI. The mean differences (range) between DBS-derived and measured plasma concentrations were 0.135 μg/mL (-0.023 to 0.327) and 0.043 μg/mL (-0.140 to 0.182) at 1- and 12-h post-dose, respectively. No significant bias in the Bland-Altman plot of the 12-h post-dose was observed, as the line of equality (zero) was within the 95% CI of the mean difference. However, at 1-h post-dose, the 95% CI did not contain zero, indicating a bias at this time point. The upper and lower limits of agreement within 95% CI at 1-h post-dose were 0.3639 μg/mL and -0.0935 μg/mL, respectively; at 12-h post-dose, these values were 0.2393 μg/mL and -0.1526 μg/mL, respectively.

### Clinical Application

**Figure 4:**
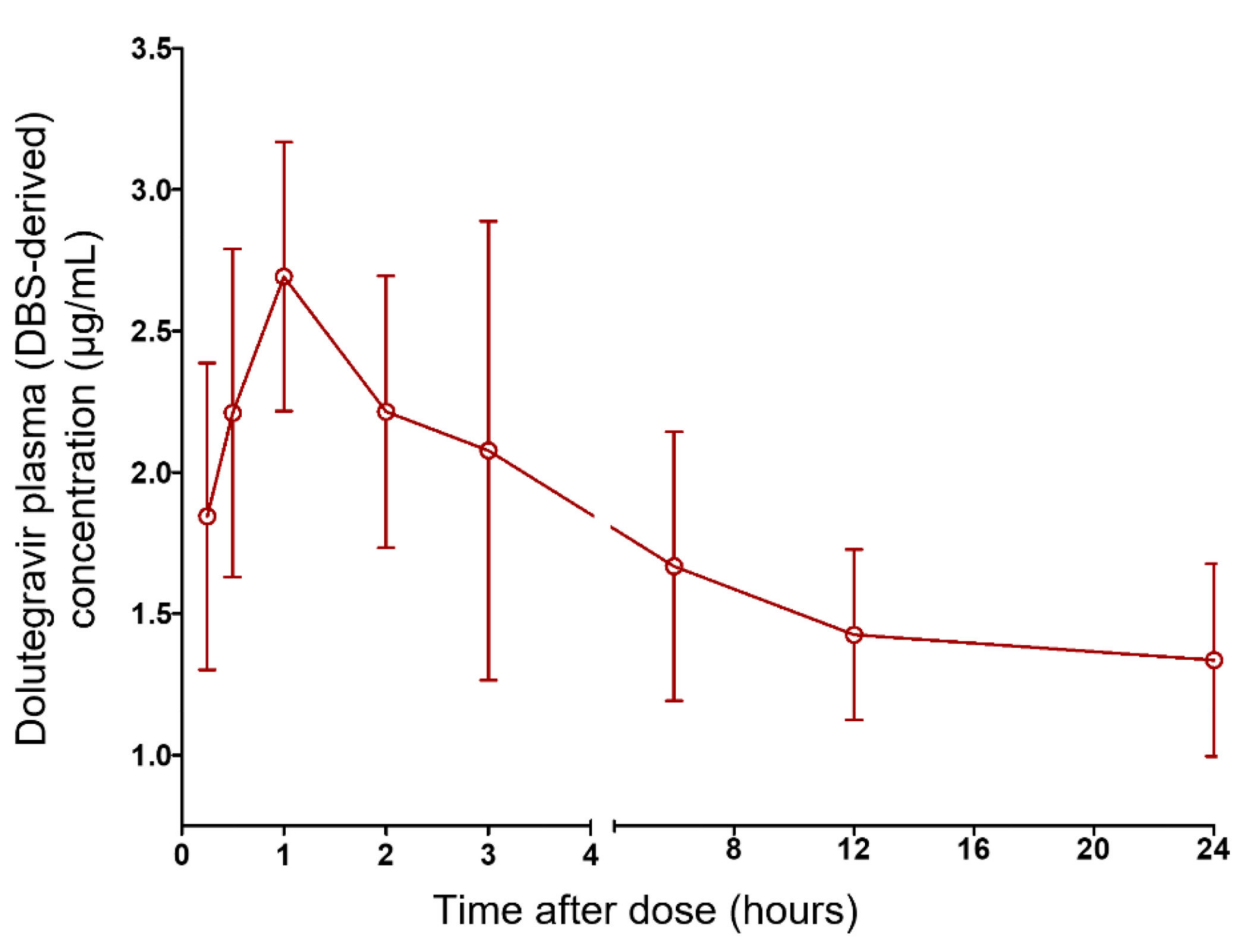
Concentration-time curve (0-24 h post-dose) of dolutegravir (mean, %CV) in plasma (DBS-derived) of HIV-positive women (n = 10) taking a regimen containing 50 mg of dolutegravir. Individual patient’s concentration-time curve is available in Fig. S1. DBS, dried blood spot; HIV, human immunodeficiency virus.

The HIV-positive women who participated in this sub-study had a mean (±SD) age of 30 years (5.94) and a weight of 61.3 kg (9.95). The women were treated with a regimen containing 50 mg dolutegravir daily for at least four weeks before pharmacokinetic sampling. All women were in the postpartum period (6 weeks to 9 months after delivery), 1 was breastfeeding, and the remaining 9 had stopped breastfeeding. The dolutegravir pharmacokinetic parameters were obtained from the DBS-derived plasma concentration-time curve (Fig. 4). Dolutegravir reached a mean (%CV) C_max_ (μg/mL) of 2.70 (24.7) at a median (range) Tmax of 1 (0.5 – 1) h. The AUC and trough concentration (C_24_) were 37.80 (23.2) μg.h/mL and 1.34 (35.6) μg/mL, respectively.

## Discussions

A simple, accurate, precise, and inexpensive method for quantifying dolutegravir in DBS using UV detection was successfully developed and validated, and its utility was demonstrated in a clinical pharmacokinetic study. To the best of our knowledge, this is the first reported method for quantifying dolutegravir in DBS. The applicability of the assay method was employed to evaluate the relationship between plasma and DBS drug concentrations, from which a full pharmacokinetic drug profile was described.

The method reported herein is expected to facilitate clinical pharmacokinetic studies of dolutegravir in settings that lack LC-MS facilities, employed by most earlier reported methods^11,27^ Additionally, the ease of sample collection, processing, transportation, and storage inherent to DBS makes the method ideal for laboratories in low-and middle-income countries, where ultra-low temperature storage facilities are either unavailable or made redundant or extremely expensive to maintain due to irregular power supply. Furthermore, the micro-volume requirement is compatible with the minimal permissible sampling volume in pediatric health research. For instance, sampling volumes ranging from 1-5% and up to 10% of total blood volume are only allowed within 24 h and over 8 weeks, respectively.^28^ Lower limits are advisable for sick children, with a maximum of 3 mL/kg post-neonatally within 24 h (3.8% of total blood volume).^28^ Therefore, the DBS technique is an appropriate approach for handling the restrictions associated with studies in this population. This is especially important as there are expectations for clinical studies in pediatric populations following dolutegravir approval in young children and the development of the drug’s pediatric formulation.^29^

The chromatographic conditions are similar to an earlier method reported for dolutegravir quantification in plasma using a UV detector and protein precipitation was used for extraction ^12^. The use of pioglitazone as an IS in this method means it cannot be used for samples from HIV patients taking pioglitazone for type 2 diabetes. However, pioglitazone is not the typical choice in HIV-related diabetes, given the risk of bladder cancer, hepatic side effects, cardiovascular morbidity, and osteoporosis.^30^ The mean percentage recovery of dolutegravir from DBS was 42.3%. Although this recovery was low, the employed extraction method afforded the most reliable and consistent results, with %CV less than 15% at all QC levels. Furthermore, changes in hematocrit values did not significantly impact the percentage recovery observed at any QC level. A lower percentage recovery is expected from DBS at low hematocrit values with partial spot analysis employed in this method, since blood spreads more than that observed at higher values. However, it has been proposed that blood with a low hematocrit contains a higher plasma proportion, possibly compensating for the lower volume obtained per punch.^31^ This has been observed in drug analytes with extremely high plasma binding such as efavirenz and some hormones.^31,32^ At efavirenz HQC, however, hematocrit levels <0.28 L/L yielded lower concentrations. Hematocrit impact on recovery depends on the nature of the interaction of the compound with blood components.^33^

In the cross-validation, the DBS concentrations underestimated plasma concentrations. This has also been observed with other drugs.^34,35^ Dolutegravir is largely bound to plasma proteins; hence, its long termination half-life of 11-14 h.^36,37^ The presence of other blood components along with plasma may explain this underestimation.^38^ However, DBS-derived plasma concentrations showed good agreement with measured plasma concentrations, as indicated by the linearity of methods and no significant bias at 12-h post-dose. Even at 1-h post-dose, the indicated bias is not clinically significant, given the wide therapeutic range of the drug and its limited propensity for drug-drug interactions and interindividual genetic variations. This could imply the potential replacement of plasma with DBS in pharmacokinetic studies. Furthermore, the upper and lower limits of agreement indicating how much higher or lower, 95% of DBS-derived plasma concentrations can vary from measured plasma concentrations, revealed that the limits were within the acceptable range, owing to the wide clinical therapeutic range and high dose forgiveness of dolutegravir.^6,39^ Hence, little or no significant clinical difference is expected when using DBS.

In the present study, dolutegravir pharmacokinetic parameters obtained from DBS-derived plasma concentrations were similar to the reported values from previous studies involving postpartum participants utilizing plasma analysis.^40,41^ The total dolutegravir exposure (37.80 μg.h/mL) obtained using the present method is higher than that observed in non-pregnant and non-breastfeeding women, consistent with earlier reports.^42^ The reported AUC values were within the predefined pharmacokinetic exposure range of 37-67 μg·h/mL and indicate adequate coverage for viral suppression during pregnancy and the postpartum period. Additionally, the C_24_ (%CV) of 1.34 (35.6) is within the predefined target (0.77-2.26 μg/mL).^6^ This observed value is higher than the minimum effective concentration of 0.064 μg/mL required to decrease the virus load by 90% (protein adjusted IC_90_), indicating the high dose forgiveness of dolutegravir.^39^ The risk of therapeutic failure in many patients is low, except in chronically nonadherent patients.

To overcome the lower sensitivity associated more with LC-UV method than LC-MS/MS, which is more sensitive to smaller samples at lower concentrations, we employed the spot stacking technique, which involved increasing the number of punches to two per spot from three spots. This technique reportedly minimizes the influence of analyte quantitation in DBS.^20^ However, a potential drawback of this approach is the reduced number of spots available for subsequent analyses if necessary. Another limitation is the lack of data on the long-term stability of dolutegravir in DBS; this is an especially important factor when envisaging clinical follow-up studies, which will necessitate maintaining samples for a while before analysis. Importantly, the long-term stability of dolutegravir in a similar dried matrix (dried breastmilk) was reported.^43^ However, establishing its stability in DBS in future investigations is desirable. In addition, the method demonstrated good clinical application in evaluating the pharmacokinetic profile of dolutegravir in 10 patients with HIV. Further application in larger pharmacokinetic studies is now needed.

## Conclusion

This method is simple, accurate, precise, and inexpensive, and its application was demonstrated in a clinical pharmacokinetic study in postpartum HIV-positive women taking regimens containing 50 mg dolutegravir daily. Its application will expand opportunities to undertake clinical pharmacokinetic studies of dolutegravir in settings with limited resources, including in pediatrics and pregnant women.

## Supplementary Material

Supplemental Data File (figure, table, etc.) - online only

## Figures and Tables

**Table 1:** Inter- and intra-day accuracy and precision and recovery of dolutegravir in dried blood spot

	Inter-assay			Intra-assay		
Nominal QCs concentrations (μg/mL)	Mean concentration (μg/mL)	Accuracy (%)	Precision (%CV)	Mean concentration (μg/mL)	Accuracy (%)	Precision (%CV)
LQC (0.50)	0.56	112.30	12.55	0.54	107.10	14.67
MQC (4.50)	3.84	85.24	8.42	4.05	90.10	3.40
HQC (8.00)	7.40	92.52	11.62	8.20	102.44	3.77

QCs, quality controls; LQC, low quality control; MQC, medium quality control; HQC, high quality control; CV, coefficient of variation.

**Table 2:** Impact of hematocrit and storage on dolutegravir recovery from dried blood spot

QC level (nominal conc., ug/mL)	HCT level (L/L)	Percentage recovery (%CV)	Actual conc. (μg /mL)	Percentage stability (%CV)
**Freshly Prepared Samples**				
LQC	0.2	43.9 (4.3)	0.57 ± 0.13	-
0.5 μg/mL	0.4	43.3 (2.1)	0.57 ± 0.11	-
	0.6	43.6 (5.9)	0.56 ± 0.12	-
MQC	0.2	39.7 (7.16)	5.17 ± 0.15	-
4.5 μg/mL	0.4	32.1 (3.7)	4.98 ± 0.11	-
	0.6	34.8 (4.9)	5.03 ± 0.13	-
HQC	0.2	36.5 (2.1)	8.31 ± 0.04	-
8.0 μg/mL	0.4	37.6 (5.4)	8.29 ± 0.04	-
	0.6	43.5 (1.7)	8.20 ± 0.02	-
**Short-term Storage (72 h at 60°C)**				
LQC	0.2	43.0 (5.3)	0.55 ± 0.10	98.8 (2.4)
0.5 μg/mL	0.4	40.3 (4.9)	0.51 ± 0.02	92.5 (2.4)
	0.6	44.0 (7.32)	0.57± 0.13	104 (2.4)
MQC	0.2	31.2 (5.7)	5.17 ± 0.15	88.8 (3.8)
4.5 μg/mL	0.4	32.7 (4.31)	5.07 ± 0.13	98.3 (0.75)
	0.6	37.7 (4.4)	4.99 ± 0.11	101 (0.96)
HQC	0.2	40.7 (3.9)	8.57 ± 0.07	97.1 (5.9)
8.0 μg/mL	0.4	41.6 (1.76)	9.19 ± 0.15	112 (0.96)
	0.6	48.7 (5.87)	9.12 ± 0.15	112 (6.4)

HCT, hematocrit; LQC, low quality control; MQC, medium quality control; HQC, high quality control; CV, coefficient of variation.
