# Supplementary figures and images for: Validation and Clinical Application of a Liquid Chromatography-Ultraviolet Detection Method to Quantify Dolutegravir in Dried Blood Spots
Source: Ther Drug Monit. Author manuscript; Available in PMC 2022 Jun 1. (PMC7612724; doi:10.1097/FTD.0000000000000929)

**FIGURE S1**


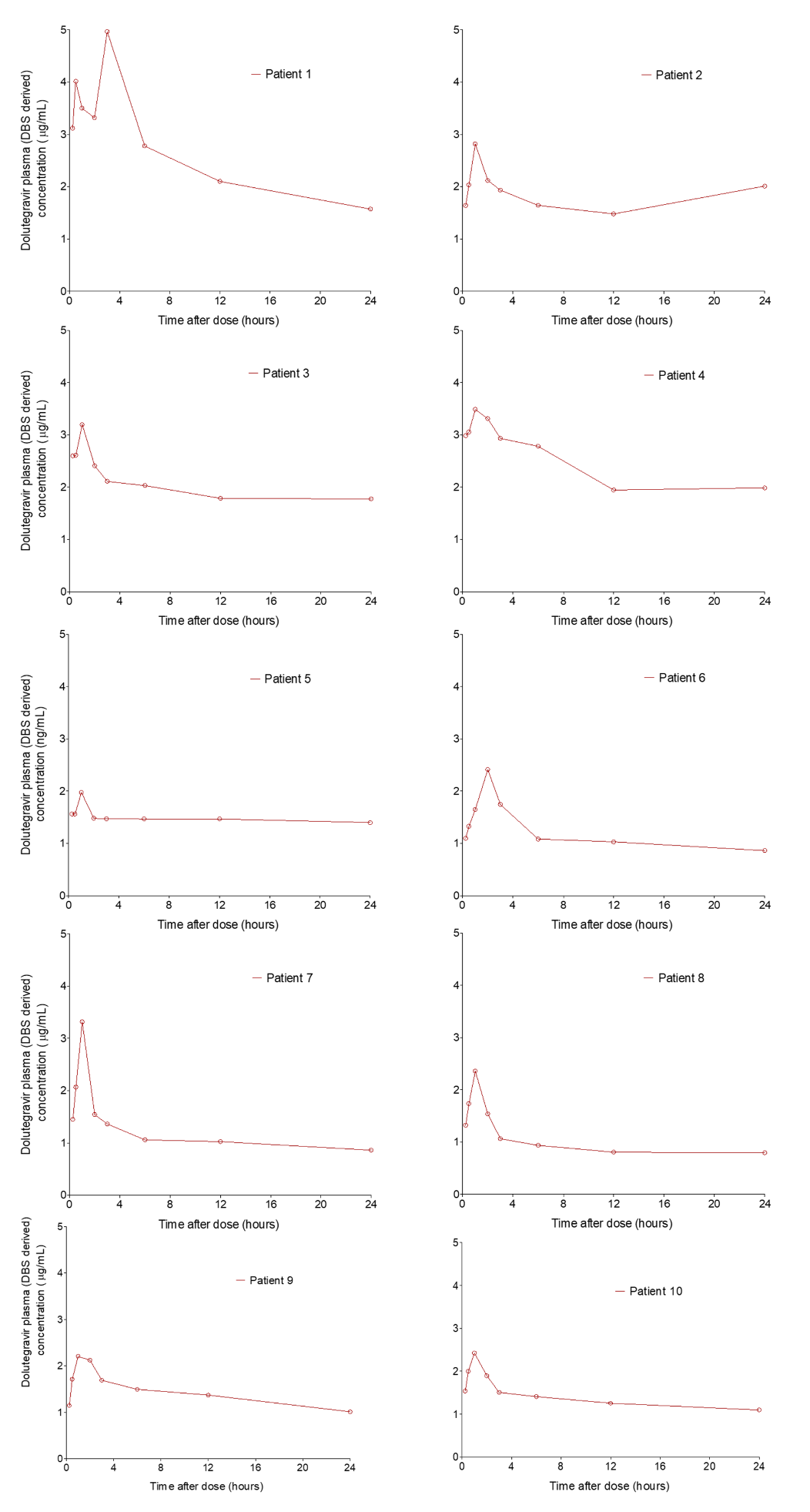

Supplement: Supplemental Data File (figure, table, etc.) - online only [file EMS136198-supplement-Supplemental_Data_File__figure__table__etc_____online_only.docx]
